# A Randomized Controlled Trial Comparing Two Infusion Doses of Dexmedetomidine on Hemodynamic Response and Extubation Quality in Neurosurgical Patients

**DOI:** 10.7759/cureus.110082

**Published:** 2026-06-01

**Authors:** Tausif Alam, Mukesh Kumar, Vishwanath Kumar, Neha Priya, Manish Kumar

**Affiliations:** 1 Anaesthesiology, M.G.M. Medical College, Jamshedpur, IND; 2 Anaesthesiology, Rajendra Institute of Medical Sciences, Ranchi, IND; 3 Community Medicine, Rajendra Institute of Medical Sciences, Ranchi, IND

**Keywords:** airway reflexes, dexmedetomidine, emergence, extubation, hemodynamic response, neurosurgery, sedation score

## Abstract

Background: Tracheal extubation is associated with significant airway and hemodynamic responses, which may be detrimental in neurosurgical patients. Dexmedetomidine, a selective α₂-adrenergic agonist, has been shown to attenuate these responses without causing respiratory depression. This study aimed to compare two infusion doses of dexmedetomidine for attenuation of stress responses during extubation.

Methods: Ninety ASA I-II patients (ages 18-65) undergoing neurosurgery were enrolled in this double-blinded, randomized controlled trial. Patients were divided into three groups: Group D1 was administered dexmedetomidine 0.4 µg/kg, Group D2 was administered 0.6 µg/kg, and Group C was administered normal saline. The study drug was infused over 20 minutes prior to extubation. Hemodynamic parameters, extubation quality, emergence time, and adverse effects were recorded and analyzed.

Results: Dexmedetomidine significantly reduced heart rate as well as mean arterial pressure compared to control during extubation and after extubation (p < 0.05). Extubation quality was significantly improved in Group D2 (1.8 ± 0.88) and Group D1 (2.5 ± 1.01) compared to Group C (3.47 ± 1.25) (p = 0.002). Sedation scores were higher in dexmedetomidine groups without respiratory compromise. Emergence and extubation times were slightly prolonged but not statistically significant. Bradycardia and hypotension were more frequent in Group D2, while breath-holding was significantly reduced in the dexmedetomidine groups.

Conclusions: Dexmedetomidine effectively attenuates extubation-related stress responses in neurosurgical patients. The 0.4 µg/kg dose provides an optimal balance between efficacy and safety, whereas the 0.6 µg/kg dose offers smoother extubation with a slightly higher incidence of manageable adverse effects.

## Introduction

Tracheal extubation is usually done towards the end of a surgery. At this time, the patient is fully conscious and can maintain and protect the airway. However, extubation is often related to several adverse airway and hemodynamic responses such as coughing, bronchospasm, laryngospasm, upper airway obstruction, hypertension, tachycardia, and dysrhythmias [1-3]. These responses can increase intracranial pressure (ICP), precipitate intracranial hemorrhage, or compromise cerebral perfusion.

These responses occur due to stimulation of the airway and sympathetic activation during emergence from anesthesia. Such complications may result in hypoventilation, pulmonary aspiration, pulmonary oedema, and myocardial ischemia in susceptible individuals [4,5]. Furthermore, sudden increases in blood pressure during extubation can lead to elevations in intraocular and intracranial pressures, which are particularly hazardous in neurosurgical patients. This may lead to wound disruption, intracranial hemorrhage, hematoma formation, brainstem herniation, postoperative deterioration in Glasgow Coma Scale (GCS), need for re-exploration of the brain, and prolonged stay in the ICU. Therefore, attenuation of extubation responses is an important objective in anesthetic management, especially in patients undergoing neurosurgical procedures [1-3]. An atraumatic extubation free of straining, laryngospasm, coughing, or patient movement can help minimize these complications [6]. Another important concern during recovery from general anesthesia is emergence delirium, which is closely associated with the anesthetic drugs administered and the duration of the anesthesia. This condition may result in adverse outcomes such as injury, hemorrhage, pain, self-extubation, and inadvertent removal of catheters [3,7]. A variety of strategies have been used to mitigate hemodynamic and airway responses during extubation, which include the use of a laryngeal mask airway at the time of emergence, extubation during a deeper plane of anesthesia, and pharmacological agents such as lignocaine, opioids, esmolol, propofol, calcium channel blockers, and magnesium sulfate [8].

Several previous studies have evaluated the highly selective α₂-agonist dexmedetomidine, reporting variable outcomes; however, it has generally been found to be beneficial in preventing extubation-related complications, including hemodynamic instability [9]. Dexmedetomidine is considered a favorable agent for attenuating the stress response to extubation due to its minimal respiratory depression, provision of easily arousable sedation, and ability to maintain hemodynamic stability. It also offers the added benefit of reducing the incidence of emergence delirium [10,11]. Various dosing regimens of dexmedetomidine have been evaluated, with intravenous bolus doses varying between 0.2 µg/kg and 1.0 µg/kg studied for their effectiveness in blunting stress responses during both extubation and intubation [12,13]. Data on the use of dexmedetomidine at extubation are sparse, especially in neurosurgical patients. Given the importance of the extubation procedure and the high incidence of associated complications, along with the need for effective preventive strategies, this study compares how two different dexmedetomidine infusion doses affect hemodynamic stability and airway responses during tracheal extubation.

The aims and objectives of this study are 1) to compare the effects of two infusion doses of dexmedetomidine on: Hemodynamic response to extubation (HR and MAP) (primary outcome), quality of extubation, time to extubate, and to emerge (secondary outcomes), and 2) to assess the incidence of adverse effects such as delayed arousal, hypotension, bradycardia, and agitation.

## Materials and methods

After getting the institutional ethics committee approval, informed consent, and registration number of this trial CTRI/2023/10/058666, we conducted a prospective, randomized, double-blind controlled study in the Department of Neurosurgery at the RIMS Ranchi over a span of one and a half years from July 2023 to Dec 2024.

Patients coming for surgery in the 18-65 years age group with ASA classification grades one and two with a supratentorial space-occupying lesion with a Glasgow Coma Scale of 15/15, who gave informed and written consent for the study, were included. While patients undergoing elective surgical procedures in the prone position, including surgery of the spine, having an allergy to dexmedetomidine, pregnant and lactating women, minors (< 18 years), and elderly patients over the age >65 years, and those who refused to give consent for the study were not included.

Sample size

The calculation was conducted by considering the study of Antony D et al. [14] using this formula: \[N = \frac{(Z_{\alpha/2} + Z_{\beta})^2 \cdot 2\sigma^2}{(\mu_1 - \mu_2)^2}\] where Z_α_/2 is the Z-value corresponding to the desired significance level (e.g.,1.96 for 0.05). Z_β_ is the Z-value corresponding to the desired power (0.84). σ is the standard deviation (SD) of the outcome. μ1−μ2 is the mean difference between groups.

Assuming a significance level (α) of 0.05 (two-tailed) corresponding to a Z value of 1.96 and a study power of 80% (β = 0.20) corresponding to a Z value of 0.84, with an estimated standard deviation (σ) of 6 and an expected mean difference (μ₁ − μ₂) of 4.6, the sample size calculated was 27 subjects in each individual group. Considering the loss to follow-up or exclusions, the groups were rounded off to 30 subjects in each group.

​Patients were divided into three groups (D1, D2, and C) using a computerized table of random numbers. Group C was administered normal saline. Group D1 was administered dexmedetomidine 0.4 mcg/kg. Group D2 was administered dexmedetomidine 0.6 mcg/kg.

Procedure

The patients having ASA grade I and II, falling in the age group 18-65 years with supratentorial space-occupying lesions, were divided into three groups randomly. Pre-anesthetic checkups, baseline investigations, and screening tests were done for all. After following the standard fasting protocol, patients were taken for surgery. On the scheduled surgery date, baseline parameters, including mean arterial pressure, heart rate (HR), blood pressure (systolic and diastolic BP), peripheral oxygen saturation (SpO₂), and continuous electrocardiogram, were recorded after attaching a monitor for multiple parameters. Peripheral venous access was established via an 18-gauge cannula in the upper limb. Patients were then administered premedication consisting of intravenous glycopyrrolate 0.004 mg/kg, midazolam 0.025 mg/kg, and fentanyl 2 µg/kg.

All patients received a standardized general anesthetic regimen. Following preoxygenation, anesthesia was induced with titrated doses of propofol, and neuromuscular blockade was achieved with vecuronium 0.1 mg/kg to provide optimal conditions for tracheal intubation. Anesthesia was sustained with isoflurane (0.6-1.0%) in a mixture of N₂O and O₂. After intubation, the patient was put on IPPV (intermittent positive pressure ventilation) with ETCO₂ targets between 30-35 mm Hg. After one hour of induction injection, paracetamol 1 gm was administered intravenously over 15 min to supplement analgesia. Test solutions were prepared by a third person not involved in the study as follows: solution D1 contained dexmedetomidine 40 µg diluted in 100 mL normal saline (concentration: 0.4 µg/mL), solution D2 contained dexmedetomidine 60 µg diluted in 100 mL normal saline (concentration: 0.6 µg/mL), and solution C consisted of 100 mL normal saline alone. Patients and researchers were blinded to the test solutions.

Just before skin closure, i.e., approximately 20 min before extubation, each patient received 1 ml/kg of the specified test solution study intravenously over 20 minutes. Isoflurane was discontinued at the last skin closure.

Vital signs, including HR, SBP, DBP, MAP, SpO₂, EtCO₂, and temperature, were monitored at baseline and at 1, 5, 10, 15, and 20 minutes following the test solution injection. Upon the return of spontaneous breathing, pharmacological reversal of residual neuromuscular blockade was performed using 50 µg/kg neostigmine and 10 µg/kg glycopyrrolate. Tracheal extubation and oropharyngeal suctioning were performed once patients met clinical criteria, specifically the ability to follow commands and maintain adequate respiration. These physiological parameters were recorded again one minute prior to extubation, during the procedure, and at 1, 5, 10, 15, 20, and 60 minutes thereafter.

The interval from the cessation of anesthetic agents to tracheal extubation was recorded as the time to extubation. During the emergence period, researchers monitored complications such as coughing, laryngospasm, delirium, and excessive sedation. Emergence time was marked as the duration from stopping anesthetic agents until the patient followed verbal commands. Clinical protocols defined hypotension as a >20% reduction in systolic blood pressure from baseline (treated with fluids or 3 mg mephentermine) and bradycardia as a heart rate below 50 bpm (treated with 0.5 mg atropine if hemodynamically unstable).

Quality of extubation was assessed based on the degree of coughing immediately after extubation using a five-point scale, where 1 = no episode of coughing; 2 = (smooth) minimum coughing, i.e., 1-2 episodes; 3 = (moderate), i.e., 3-4 episodes; 4 = (severe) 5-10 episodes; 5 = (poor) laryngospasm or >10 episodes of coughing [13].

Postoperative sedation was evaluated on arrival in the post-anesthesia care unit (PACU) using the six-point Ramsay Sedation Scale: 1 = having anxiety, agitation, or restlessness; 2 = having orientation, cooperation, and calmness; 3 = having drowsiness yet responsive to commands; 4 = sleepy but demonstrates a prompt response to slight glabellar tapping or loud sound; 5 = sleepy and exhibits a delayed response to slight glabellar tapping or loud sound; 6 = nonresponsive [15].

Data collection and statistical analysis: Data were stored in MS Excel (Redmond, USA), and analysis was done with IBM Corp. Released 2016. IBM SPSS Statistics for Windows, Version 22. Armonk, NY: IBM Corp. Continuous type variables were represented as mean ± standard deviation and evaluated via ANOVA. Categorical variables were presented as frequencies or percentages and interpreted utilizing the Chi-square (χ²) test. Statistical significance was set as p < 0.05.​​​​​​​

## Results

Demographic and baseline characteristics

Ninety ASA Grade I or II patients, aged 18 to 65 and scheduled for neurosurgery, were recruited for the study. Table 1 describes demographic characteristics. The perioperative variables are summarized in Table 2.

**Table 1:** Demographic characteristics of the patients under study *One-way ANOVA was used to compare the mean and standard deviation (F-value). Statistical significance was set at p < 0.05. A ^chi-square test was applied for sex and ASA grade (chi-square). ASA: American Society of Anesthesiologists

Variables	Group C (Mean ± SD)	Group D1 (Mean ± SD)	Group D2 (Mean ± SD)	F-value/Chi square value	p-value
Age (years)	37.37 ± 7.04	37.53 ± 9.70	38.83 ±12.0	0.17*	0.844
Weight (kg)	56.7 ± 5.20	55.7 ± 3.97	56.6± 4.68	0.55*	0.581
Sex (M/F)	15/15	13/17	13/17	0.358^	0.836
ASA Grade (I/II)	14/16	15/15	14/16	0.089^	0.956
Total	30	30	30	-	-

**Table 2:** Perioperative and recovery characteristics of the patients under study One-way ANOVA was used to compare the means and standard deviations. statistical significance was set at p-value < 0.05.

Variables	Group C (Mean ± SD)	Group D1 (Mean ± SD)	Group D2 (Mean ± SD)	F-value	p-value
Duration of surgery (min)	117.73 ± 3.72	119 ± 2.53	115.6 ± 19.80	0.63	0.245
Duration of Anesthesia (min)	135.6 ± 4.05	136.8 ± 3.04	137.57 ± 2.87	1.74	0.181
Time to Extubate	11.77 ± 1.695	12.9 ± 1.80	15.57 ± 1.94	2.3	0.107
Emergence Time	8.2 ± 2.24	9.7 ± 1.95	12.53 ± 2.09	2.2	0.121

On comparing the three study groups, they were uniform in terms of their weight, age group, and sex distribution. The mean age in Groups C, D1, and D2 was 37.37 ± 7.04, 37.53 ± 9.70, and 38.83 ± 12.0 years, respectively. Mean body weight was 56.7 ± 5.20 kg in Group C, 55.7 ± 3.97 kg in Group D1, and 56.6 ± 4.68 kg in Group D2. Using one-way ANOVA, an insignificant difference was seen in age (p = 0.844) or weight (p = 0.581). Gender distribution in the 3 groups was comparable (χ² test, p = 0.836). On statistical analysis using Χ2 test, the variable (ASA grading) of the three groups was comparable with the p-value of 0.956. Hence, ASA grading was not a significant variable in this study (Table 1).

At the completion of surgery, the test solution was initiated at the onset of skin suturing and continued for 20 minutes, following which extubation was performed. Hemodynamic parameters were monitored during this period, immediately after extubation, and at 1 hour postoperatively, and were analyzed using one-way ANOVA.

One-way ANOVA revealed no significant difference between the three groups regarding surgical duration (p = 0.245) or anesthesia time (p = 0.181), confirming group comparability. While the mean time to extubation was longer in the D2 group (15.57 ± 1.94 min) compared to the D1 (12.9 ± 1.82 min) and C (11.77 ± 1.69 min) groups, this difference did not reach statistical significance (p = 0.107). Time to extubation between the three groups was comparable (Table 2). The mean emergence time also followed a similar trend (p = 0.121), with longer emergence times observed in Groups D2 compared to Groups D1 and C, which was statistically nonsignificant.

Hemodynamic parameters

**Figure 1:**
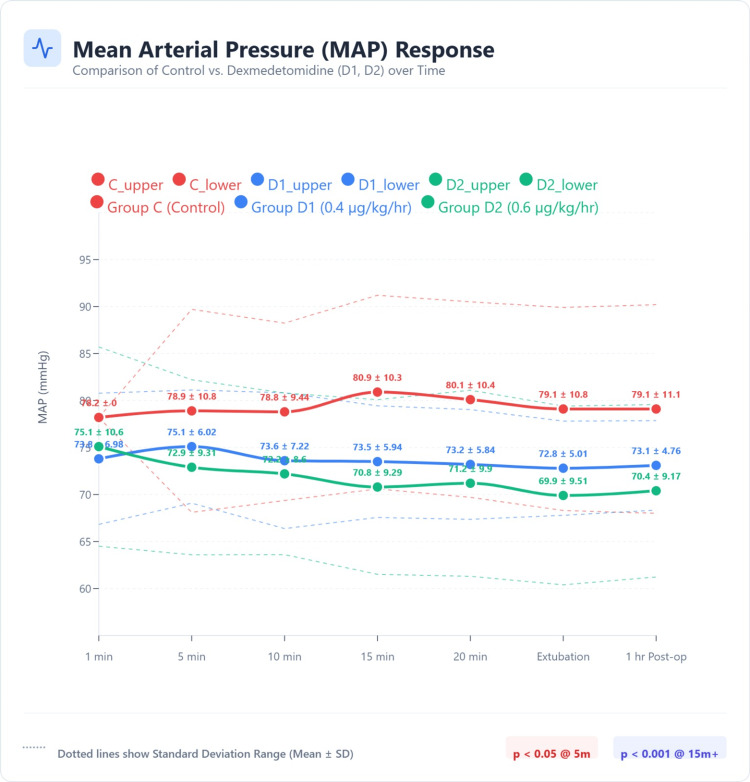
Analysis of mean arterial pressure of groups C, D1 and D2 One-way ANOVA was used to compare means and deviations. Statistical significance was set at p<0.05; high significance was set at p<0.001.

Figure 1 shows the statistical analysis of MAP in the 3 study groups. Baseline MAP values at 1 minute were comparable among Groups C, D1, and D2 (78.2, 73.8, and 75.1 mmHg, respectively). A gradual decline in MAP was observed in all groups; the reduction was more pronounced in Groups D1 and D2. At 5 minutes, this reduction was significant in Group D2 than in Group C (P = 0.012). From 10 minutes until post-extubation and up to 1 hour postoperatively, MAP differences among the groups were highly significant (p < 0.001), with Groups D1 and D2 consistently showing lower values than Group C.

**Figure 2:**
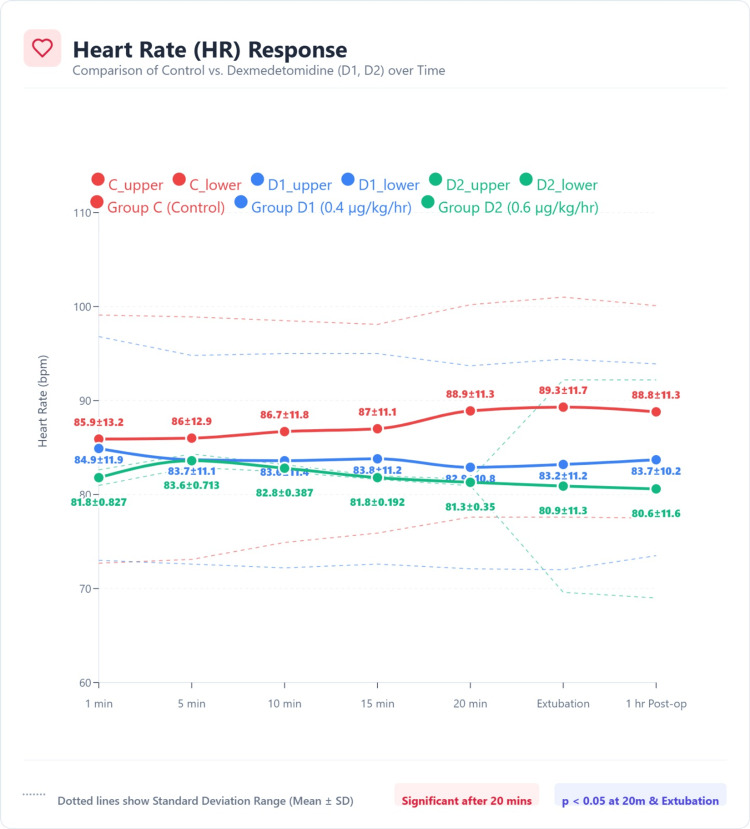
Analysis of heart rate of groups C, D1, and D2 One-way ANOVA was used to compare means and standard deviations. Statistical significance was set at p<0.05.

Analysis of heart rate at different time intervals demonstrated the mean values across the three groups, as shown in Figure 2. A statistically significant difference was observed 20 minutes after initiation of the test solution, which persisted after extubation and up to 1 hour postoperatively (p = 0.035, 0.016, and 0.018, respectively).

Similar trends were observed for systolic and diastolic blood pressure, with Groups D1 and D2 demonstrating attenuated hemodynamic responses compared to Group C throughout the observation period.

Respiratory parameters

Oxygen saturation (SpO₂) and EtCO₂ values remained stable and comparable across all groups at all measured time points. No statistically significant differences were noted in SpO₂ or EtCO₂ values among Groups C, D1, and D2 (p > 0.05), indicating that the study drugs did not adversely affect respiratory function.

Overall, dexmedetomidine infusion at both 0.4 µg/kg and 0.6 µg/kg effectively attenuated hemodynamic responses without compromising respiratory parameters during and after neurosurgical procedures.

Extubation quality and sedation

5-Point Extubation Score

The mean 5-point extubation score was 3.47 ± 1.25 in Group C, 2.50 ± 1.01 in Group D1, and 1.80 ± 0.88 in Group D2 (Table 3). One-way ANOVA demonstrated a statistically significant difference among the groups (p = 0.002). Group D2 demonstrated the lowest mean score, indicating the smoothest extubation, while Group C had the highest score.

**Table 3:** Comparison of 5-point scores and Ramsay sedation scores of groups C, D1, and D2 One-way ANOVA was used for comparing means and standard deviation. Statistical significance was set at a *p-value <0.05.

Extubation quality variable	Group C (Mean ± SD)	Group D1 (Mean ± SD)	Group D2 (Mean ± SD)	F-value	p-value
5 Point Score	3.47±1.25	2.5±1.008	1.8±0.88	23.4	0.002*
Ramsay Scale	1.7 ±0.70	2.6±1.22	2.93± 1.14	9.1	0.003*

Sedation (Ramsay Sedation Scale)

The mean Ramsay Sedation Scale score was 1.70 ± 0.70 in Group C, 2.60 ± 1.22 in Group D1, and 2.93 ± 1.14 in Group D2 (Table 3). It was revealed statistically that a notable difference exists between the groups under study (p = 0.003). Sedation was highest in Group D2 and lowest in Group C. These findings indicate that dexmedetomidine infusion, particularly at 0.6 µg/kg (Group D2), improves extubation quality while providing moderate sedation without respiratory compromise.

The incidence of cardiovascular and other side effects among the three groups is summarized in Table 4. Bradycardia only occurred in three (10%) patients of Group D1 and four (13.33%) patients of D2, while Group C had no events (p=0.133). While dexmedetomidine infusion appeared to lower the risk of hypertensive episodes, it was associated with isolated cases of hypotension in four patients (13.33%) in D1 and five patients (16.66%) in D2. However, these adverse effects were not statistically significant compared to group C (p = 0.232).

**Table 4:** Comparison of complications among the study groups Chi-square test was used to compare the frequency and percentage of variables. Statistical significance was set at *p-value <0.05.

Complications	Group C N (%)	Group D1 N (%)	Group D2 N (%)	Chi-square value	p-value
Bradycardia	0 (0)	3 (10)	4 (13.33)	4.03	0.133
Hypotension	1 (3.33)	4 (13.33)	5 (16.66)	2.92	0.232
Breath holding	5 (16.66)	1 (3.33)	0 (0)	7.58	0.023*
Nausea and vomiting	5 (16.66)	3 (10)	4 (13.33)	0.58	0.750

Breath-holding was observed in five (16.6%) patients in group C, only 1 (3.33%) patient in group D1, and none of the patients in the last group D2 (p = 0.023).

Nausea occurred in all the groups. The combination of nausea and vomiting was seen in five (16.66%) patients in Group C, three (10%) patients in Group D1, and four (13.33%) patients in Group D2. Isolated vomiting was noted only in two (6.6%) patients in Group C, with no occurrences in Groups D1 or D2. These findings indicate that postoperative nausea and vomiting were generally mild and more frequent in Group D2, while dexmedetomidine at the lower dose (Group D1) was associated with the lowest incidence of such complications.

## Discussion

Hemodynamic responses during intubation and extubation in neurosurgical patients are complex, with risks including tachycardia, hypertension, increased intracranial pressure, bleeding, airway complications, and postoperative nausea and vomiting. These may result from pain, catecholamine surges, or delayed emergence from anesthesia. Maintaining stable hemodynamics requires meticulous monitoring, advanced airway management, and coordinated perioperative care.

Pharmacological interventions such as dexmedetomidine, a selective α2-adrenoceptor agonist, have been used to attenuate these stress responses. Dexmedetomidine provides analgesic, sedative, amnestic, anxiolytic, and sympatholytic effects without significant respiratory depression. Decreasing norepinephrine release, it reduces heart rate and mean arterial pressure while also decreasing anesthetic and opioid requirements. Its eightfold greater selectivity for α2 receptors compared to clonidine enhances its efficacy as an anesthetic adjuvant [9].

This study compared two continuous infusion dosing regimens of dexmedetomidine (0.4 µg/kg/hr and 0.6 µg/kg/hr) with placebo in neurosurgical patients to evaluate their effect on hemodynamic stability during extubation and recovery. Demographic variables were comparable among groups.

Both dexmedetomidine doses effectively attenuated hemodynamic responses without causing a delay in emergence from anesthesia, as evidenced by lower heart rates and mean arterial pressures before and during extubation, maintained up to one hour postoperatively. Oxygen saturation, EtCO₂, and body temperature remained unaffected at both doses, indicating respiratory safety.

Recovery parameters showed that time to extubation and emergence time both were insignificantly increased in the dexmedetomidine groups compared to placebo. The 5-point extubation score indicated smoother extubation in dexmedetomidine groups, particularly in the higher dose group. Ramsay Sedation Scale scores were higher in dexmedetomidine groups, with the highest sedation observed in the 0.6 µg/kg/hr group.

Cardiovascular complications were minimal. Bradycardia and hypotension occurred infrequently in the higher dose dexmedetomidine group. Postoperative nausea and vomiting were mild, with comparable incidence in the three groups.

In patients with chronic hypertension, the autoregulatory range for cerebral and renal perfusion is typically shifted to higher levels; therefore, maintaining mean arterial pressure within an appropriate range is of critical importance [16]. Vital organs, including the brain and kidneys, are particularly susceptible to hypoperfusion during sudden and rapid decreases in blood pressure. However, in our study, none of the patients experienced a mean arterial pressure below 65 mmHg, thereby preserving autoregulation, consistent with the results of Liyakhath A et al. [17].

Our findings are consistent with previous studies. Turan G et al. [13] reported stable hemodynamics and smoother extubation with dexmedetomidine infusion in neurosurgical patients. Comparable findings have been reported in another study, where an infusion of dexmedetomidine at 0.6 μg/kg/h significantly reduced blood pressure without causing any notable respiratory depression [18]. Similar results were yielded by the study conducted by Talke P et al. [19].

The Ramsay Sedation score in our study was not significant, as also shown in the study by Lu Q et al. [15]. In this study, three doses of the drug: low, medium, and high, were tested in neurosurgery patients, which demonstrated that there was no notable difference between the medium- and high- dose of the drug. Our study did not reveal any significant variations between the groups while analyzing oxygen saturation. This result was similar to a study done by Ard JL et al. [20], which described the effects of dexmedetomidine in awake patients during craniotomy.

The 5-point scoring system used to assess the quality of extubation demonstrated the lowest scores in the D2 group (0.6 mcg/kg). This finding aligns with the observations of Chaudhary M et al. [21] in 2021, which evaluated two dose variations of dexmedetomidine (0.3 mcg/kg and 0.5 mcg/kg). Their study reported that the 0.5 mcg/kg dose was better tolerated during extubation and was associated with smoother extubation with minimal coughing.

Emergence time and time to extubation showed similarity in the control and lower dose groups, but a slight and non-significant increase in the higher dose group. These findings are consistent with the study by Antony D et al. [14], which compared two doses of dexmedetomidine (0.5 and 1 mcg/kg) and reported that the lower dose provided better control of the stress response during extubation without affecting emergence time. Similar observations were reported by Aksu E et al. [11] in patients undergoing rhinoplasty, where the lower dose was found to maintain better hemodynamic stability without adversely affecting recovery.

Limitations

While providing valuable insights, this single-center study’s small sample size warrants further validation in larger, multi-center trials. Only two dexmedetomidine infusion doses were evaluated; intermediate doses were not studied. Postoperative complications were monitored for only 1 hour; delayed complications beyond this period were not assessed. Depth of anesthesia was not objectively monitored using BIS or other modalities, which could affect hemodynamic interpretation.

## Conclusions

Dexmedetomidine infusion at 0.4 µg/kg/hr effectively attenuates hemodynamic fluctuations and stress responses during extubation in neurosurgical patients, with minimal cardiovascular or respiratory complications. While the higher dose (0.6 µg/kg/hr) also stabilizes hemodynamics, it may be associated with a slightly increased incidence of bradycardia or hypotension. Therefore, the lower dose provides an optimal balance of safety and efficacy for smooth extubation.
